# The Influence of h-BN Distribution Behavior on the Electrothermal Properties of Bismaleimide Resin

**DOI:** 10.3390/polym17141929

**Published:** 2025-07-14

**Authors:** Weizhuo Li, Xuan Wang, Mingzhe Qu, Xiaoming Wang, Jiahao Shi

**Affiliations:** 1 School of Intelligence and Civil Engineering, Harbin University, Harbin 150076, China; 2 Key Laboratory of Engineering Dielectrics and Application, Ministry of Education, Harbin University of Science and Technology, Harbin 150080, China; 3 School of Material Science and Chemical Engineering, Harbin University of Science and Technology, Harbin 150080, China

**Keywords:** bismaleimide, h-BN, thermal conductivity, Y. Agari model

## Abstract

Thermal conductive composite materials have excellent electrical insulation properties, low cost, and are lightweight, making them a promising alternative to traditional electronic packaging materials and enhancing the heat dissipation of integrated circuits. Due to the differences in specific surface area and volume, thermal conductive fillers have poor interface connections between the polymer and/or thermal conductive filler, thereby increasing phonon scattering and affecting thermal conductivity. This article uses bismaleimide resin as the matrix and h-BN as the thermal conductive filler. The evolution laws of thermal conductivity and dielectric properties of thermal conductive composite materials were systematically characterized through multi-scale filler control and gradient filling design. Among them, h-BN with a diameter of 10 μm has the most significant improvement in thermal conductivity. When the filling amount is 40 wt%, the thermal conductivity reaches 1.31 W/(m·K).

## 1. Introduction

Thermal conductive materials are widely used in various areas of national defense and the economy, especially in the fields of microelectronic packaging and component heat dissipation. In recent years, with the rapid development of science and technology, and the rise of industries such as 5G communication, new energy, and intelligent manufacturing, electronic devices have rapidly developed towards high integration, multifunctionality, and miniaturization [1,2,3]. The power density and heat output of electronic components calculated per unit volume has also significantly increased, making it difficult to dissipate the generated heat in a timely and effective manner, thereby reducing the performance, effective life, and safety stability of the equipment. It has become one of the key factors limiting the further development of high power and high integration in the field of electronic communication. The key to solving these problems is to develop electronic packaging materials with high thermal conductivity, and developing high thermal conductivity, low-cost, and environmentally friendly preparation methods is also a challenge [4,5,6].

The ideal thermal conductive electronic packaging material should not only have high thermal conductivity, but also excellent thermal stability and electrical insulation. Although traditional thermal conductivity materials such as metals and inorganic carbon materials can meet the requirements for use, they have disadvantages such as high density, difficulty in processing, susceptibility to chemical corrosion, and poor mechanical properties. Moreover, they generally have conductivity and can only be applied in fields that do not require high electrical insulation and breakdown voltage [7,8,9]. Polymer materials have become one of the candidate materials in the field of electronic device heat dissipation due to their excellent properties such as chemical stability, electrical insulation, low density, easy processing, light weight, and low cost [10]. However, the amorphous structure and vibration of large molecular chains result in low thermal conductivity (0.1–0.5 W/(m·K)) for the vast majority of pure polymers, severely limiting their application in this field and making it difficult to directly meet the efficient heat dissipation requirements of integrated electronic devices [11,12,13]. Therefore, it is imperative to develop polymer-based composite materials that are thermally conductive but insulating. Further research has found that poor interface contact between thermal conductive fillers and polymers can increase phonon scattering and affect thermal conductivity [14,15,16]. The increase in thermal conductivity can only be achieved at a higher addition amount, but it inevitably reduces other properties of the material [17,18,19]. Therefore, studying the distribution behavior of thermal conductive fillers on the thermal conductivity of composite materials can fully leverage the high inherent thermal conductivity of thermal conductive fillers. It is of great significance to improve the comprehensive performance of thermal conductive composite materials.

This article uses bismaleimide resin as the matrix and h-BN as the thermal conductive filler. Through multi-scale filler control and gradient filling design, the synergistic evolution law of thermal and electrical properties of thermal conductive composite materials is systematically characterized. The Y. Agari thermal conductivity model is used to describe the efficiency of the thermal conductivity pathway construction of fillers in thermal conductive composite materials, clarify the influence mechanism of filler size and mass fraction on phonon transmission efficiency of thermal conductive composite materials, and screen the best h-BN filler scheme that meets the synergistic optimization of high thermal conductivity and high insulation.

## 2. Experimental

### 2.1. Materials

Diphenylmethane type bismaleimide (BMI) was purchased from Shandong Minghoude Polymer Materials Co., Ltd., Binzhou, China; Ortho diallyl bisphenol A (DBA) was purchased from Shandong Laiyu Chemical Co., Ltd., Laizhou, China; Hexagonal boron nitride (h-BN) was purchased from Shanghai Macklin Biochemical Co., Ltd., Shanghai, China; Tert-Butyl peroxybenzoate (TBPB) was purchased from Shanghai Macklin Biochemical Co., Ltd., Shanghai, China; N,N-dimethylacrylamide (DMAA) was purchased from Jinan Yuyi Chemical Co., Ltd., Jinan, China.

### 2.2. Preparation of BMI-DBA/DMAA/h-BN Composite Material

BMI and DBA is placed in a 1:1 molar ratio into a three-necked flask and heated in a 135 °C oil bath for 25 min. After the reaction is complete, the temperature is lowered to 50 °C. A 30 wt% DMMA diluent containing BMI and DBA total mass is added, and the mixture is stirred at 50 °C for 1 h. After stirring evenly, the mixture is poured out into a material bottle to obtain bismaleimide resin gel.

h-BN was added to BMI-DBA/DMAA adhesive solution at certain mass fractions (10 wt%, 20 wt%, 30 wt%, and 40 wt%) and dispersed in a high-speed disperser for 6 h to obtain BMI-DBA/DMAA/h-BN composite adhesive solution. BMI-DBA/DMAA/h-BN and TBPB are put into a three-necked bottle in a mass ratio of 100:1 and dispersed under vacuum in an oil bath at a constant temperature of 80 °C for 30 min. After being evenly dispersed, it is poured into a polytetrafluoroethylene mold and placed in an oven for curing. The curing temperature program is set to 135 °C/2 h + 160 °C/2 h + 200 °C/2 h + 230 °C/4 h. In this work, three different scales of h-BN with sheet diameters of 500 nm, 1 μm, and 10 μm were used as thermal conductive fillers to prepare BMI-DBA/DMAA/h-BN composite adhesives with different compositions.

### 2.3. Characterization

The dielectric properties of the material were tested using the Alpha-A broadband dielectric spectrometer produced by Novocontrol in Germany. Based on the theory of dielectric polarization, the dielectric properties of materials and their changes with frequency are measured. The measurement range of conventional power frequency complex capacitance and dielectric loss factor is extended to the low-frequency and high-frequency regions, which can directly obtain the dielectric loss factor and relative dielectric constant of materials. The thickness of the sample is 1 mm, the electrode diameter is 20 mm, the voltage at both ends of the sample is 1 V, and the test temperature is set to 25 °C. The relative dielectric constant and dielectric loss factor (tan δ) of the material in the frequency range of 1–10^6^ Hz are characterized for the test sample.

The conductivity current was tested using a self-made three electrode testing system. The test field strength is set to 10 kV/mm, the test frequency is 50 Hz, the test temperature is selected as 25–150 °C, the step size is 25 °C, and each temperature point is maintained for 30 min before measurement. Calculate the AC conductivity using Equation (1) as follows:

*J* = *γ_v_*
*E* (1)

where *E* represents the applied field strength (V/m) for testing, and *J* represents the conductivity current density (A/m^2^). *γ_v_* represents conductivity.

The uniform electric field of the column electrode is used for AC breakdown testing at power frequency, and the thickness of the test sample is 1 mm. The sample is placed between two electrodes and the electrodes are placed in a container containing transformer insulation oil. The pressurization method used during the testing process is a uniform pressurization with a pressurization rate of 1 kV/s. Ten effective tests are conducted on the same sample for each measurement, and the relevant data are recorded. Probability statistics are performed using a two parameter Weibull distribution, and the Weibull distribution failure probability density distribution is calculated using Equation (2) [20,21]:

(2) $$P(E)=1-exp[-{\left(\frac{E}{Eb}\right)}^{\beta }]$$

where P(*E*) is used to represent the cumulative failure probability, *E* represents the breakdown field strength, *Eb* represents the characteristic breakdown field strength obtained by fitting the two parameter Weibull distribution when the cumulative failure probability is 63.2%, and *β* represents the shape parameter.

The LFA447 laser thermal conductivity meter produced by the German bavaria company Netzsch (Selb, Germany) was used to test the thermal conductivity of composite materials,. The test sample has a diameter of 12.7 mm and a thickness of 1 mm.

The viscosity of the adhesive was tested using the VR3000 from TQC Sheen company in Rotterdam, The Netherlands.

## 3. Results and Discussion

### 3.1. Synthesis of BMI-DBA/DMAA

**Figure 1:**
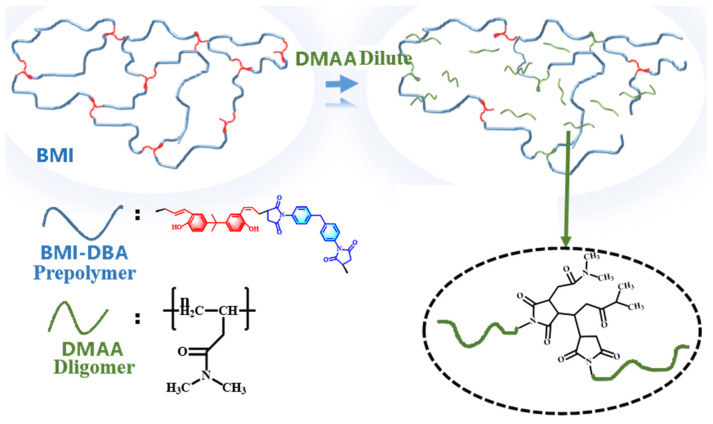
BMI-DBA/DMAA synthesis route.

The synthesis route of BMI-DBA/DMAA composite material is shown in Figure 1. Compared to BMI resin, DMAA is a single functional monomer that forms several linear molecular chains during the heating reaction. The majority of these linear molecular chains act as “bridging agents” to participate in the crosslinking reaction of BMI resin, while a small portion is physically entangled in the crosslinking system in the form of an interpenetrating network, increasing the steric hindrance of the system and thereby reducing the crosslinking density of BMI resin system. Therefore, the introduction of DMAA will reduce the crosslinking density of BMI resin, thereby improving the flexibility of BMI resin.

### 3.2. Thermal Conductivity Characteristics

To investigate the influence of h-BN distribution behavior on the thermal conductivity of BMI-DBA/DMAA, BMI-DBA/DMAA with different scales of h-BN were prepared at 10 wt%, 20 wt%, 30 wt%, and 40 wt% filling levels, and the thermal conductivity and viscosity (80 °C) of the above samples were tested. The test results are shown in Table 1.

**Figure 2:**
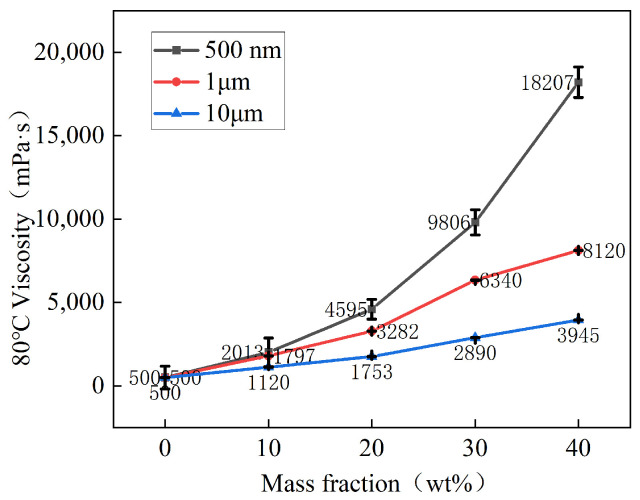
The viscosity curve of BMI-DBA/DMAA/h-BN composites at 80 °C.

A line graph was drawn based on the viscosity of BMI-DBA/DMAA/h-BN thermal conductive composite materials with different h-BN distribution patterns in Table 1, as shown in Figure 2. According to Table 1 and Figure 2, at 80 °C, the viscosity of the adhesive solution with 40 wt% of h-BN with a diameter of 500 nm increased from 980 mPa·s to 18,207 mPa·s. However, at a dosage of 40 wt%, the viscosity of the filled h-BN thermal conductive potting adhesive with a diameter of 1 μm and 10 μm increased from 980 mPa·s to 8120 mPa·s and 3945 mPa·s, respectively, at 80 °C. From the Figure 2, it can be seen that h-BN with smaller particle size has a greater thickening effect on the adhesive solution. This is because when the particle size of the powder is small, the area of direct contact between the particle surfaces is relatively large, resulting in an increase in friction between particles and an increase in viscosity. The viscosity of thermal conductive resin adhesive is crucial to its thermal conductivity. If the viscosity of the adhesive is too high, it will make it difficult for the thermal conductive adhesive to penetrate and effectively perform functions such as heat dissipation and insulation protection.

**Figure 3:**
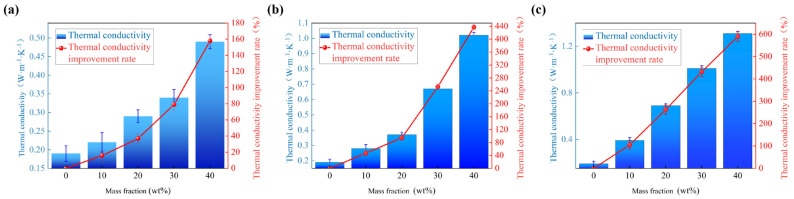
The thermal conductivity and the improvement of thermal conductivity of BMI-DBA/DMAA/h-BN. (**a**) 500 nm; (**b**) 1 μm; (**c**) 10 μm.

According to the different h-BN distribution patterns in the table, line charts and bar charts were drawn for the BMI-DBA/DMAA thermal conductivity and improvement rate, as shown in Figure 3.

According to Figure 3, the thermal conductivity of BMI-DBA/DMAA/h-BN increases with the increase of h-BN content. The thermal conductivity of h-BN thermal conductive composite material with a diameter of 500 nm increases from 0.19 W/(m·K) to 0.49 W/(m·K), with an increase rate of 157.89%. The thermal conductivity of h-BN thermal conductive composite material with a diameter of 1 μm increases from 0.19 W/(m·K) to 1.02 W/(m·K), with an increase rate of 436.84%. The thermal conductivity of h-BN thermal conductive composite material with a diameter of 10 μm increases from 0.19 W/(m·K) to 1.31 W/(m·K), with an increase rate of 589.47%. It can be seen that the larger the diameter of the h-BN-filled thermal conductive composite material, the greater the improvement rate of thermal conductivity. This is because when the particle size of the thermal conductive filler increases, the path of heat conduction in the material becomes shorter, and the resistance to conduction decreases. The anisotropic morphology of h-BN sheet materials is such that large-sized h-BN can provide a larger contact area and a more effective thermal conductivity path, thereby improving thermal conductivity.

Analyzing the influencing factors of material thermal conductivity from a theoretical perspective and establishing an equation to describe the thermal conductivity of materials is of great significance for designing materials with specific thermal conductivity and predicting their thermal conductivity. Under high filling conditions, the Y. Agari model considers the influence of the filler thermal conductivity network and filler introduction on matrix morphology. This model is a “semi empirical” thermal conductivity model that can be fitted based on experimental data. Therefore, after a large amount of experimental data validation, the parameters of this model can become increasingly accurate [22,23]. As shown in Equation (3):

lg*λ_c_* = *V_f_C*_2_lg*λ_f_* + (1 − *V_f_*)lg(*C*_1_*λ_p_*) (3)

where *C*_1_ represents the crystallinity of the polymer; *C*_2_ is the efficiency of constructing the thermal conduction pathway; *λ_p_* is the thermal conductivity of the polymer matrix (0.32 W/(m·K)); *λ_c_* is the thermal conductivity coefficient of the thermal conductive composites; *V_f_* is the volume fraction of the filler in the thermal conductive sealant; *λ_f_* is the thermal conductivity of the filler (57.6 W/(m·K)).

**Figure 4:**
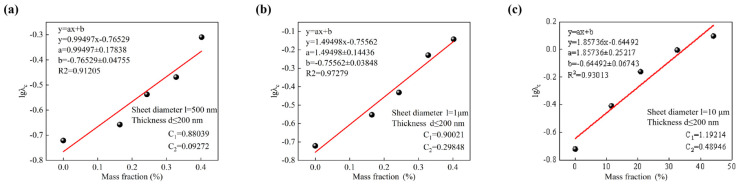
Fitting results of BMI-DBA/DMAA/h-BN composites and the Y. Agari thermal conductivity model.(**a**) 500 nm; (**b**) 1 μm; (**c**) 10 μm.

By testing the thermal conductivity of BMI-DBA/DMAA/h-BN with added particle sizes of 500 nm, 1 μm, and 10 μm h-BN at different filling amounts, and replacing the mass fraction with the corresponding *V_f_*, the construction efficiency was obtained according to the Y. Agari model, and the results are shown in Figure 4. After fitting and calculating the composite material using the Y. Agari model, the results are shown in Table 2.

As shown in Figure 4 and Table 2, the C_2_ values are 0.093, 0.156, and 0.489, respectively. This indicates that it is easier to construct a thermal conductivity path in the BMI-DBA/DMAA resin system by filling h-BN with a diameter of 10 microns, and its C_2_ value is relatively high. This difference is mainly attributed to the “ordered” and “disordered” distribution of h-BN of different sizes in the resin. In the BMI-DBA/DMAA/h-BN system, the efficiency of constructing thermal conductivity paths using micrometer scale h-BN is significantly higher than that of nanometer scale h-BN. Its core lies in the fact that 10-micron h-BN has a larger mean free path during phonon conduction, which can better avoid phonon scattering. It has obvious advantages in constructing thermal conductivity paths, which is consistent with the thermal conductivity data in composite materials.

**Figure 5:**
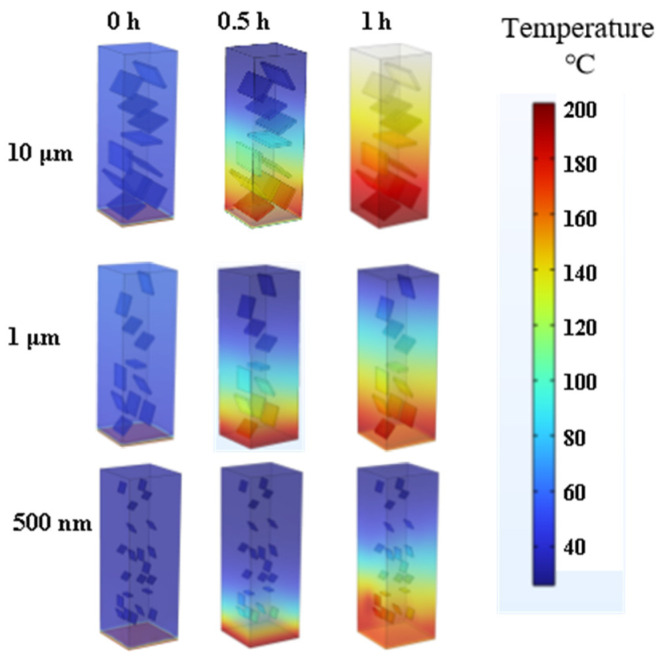
Finite element simulation of heat transfer in BMI-DBA/DMAA/h-BN composites.

By using finite element thermal field analysis, the heat transfer ability of thermal conductive composite materials can be presented to a certain extent, and based on this, the difficulty of constructing thermal conductive pathways with different sizes of fillers can be analyzed. In the simulation process, a two-dimensional cross-section of BMI-DBA/DMAA/h-BN block material was selected, with a bottom width of 30 mm and a block height of 100 mm. A heat source of 200 °C was set at the bottom of the block, and the initial temperature of the material was 25 °C, with thermal insulation around it. This section compares the thermal field distribution of BMI-DBA/DMAA/h-BN prepared from h-BN with different diameters, as shown in Figure 5. The dispersion of h-BN with different diameters in BMI-DBA/DMAA exhibits a disordered and disordered dispersion pattern. However, due to the settling phenomenon during the curing process, h-BN accumulates at the bottom of the resin, resulting in a significant decrease in the efficiency of h-BN forming a thermal conductivity pathway in the thermal conductive composite material. In terms of filler size, the efficiency of constructing thermal conductivity pathways using nano-sized h-BN is low, while the efficiency of constructing thermal conductivity pathways using larger sized h-BN fillers is much higher. The thermal conductivity of BMI-DBA/DMAA/h-BN prepared from large diameter h-BN is superior to that of thermal conductive composite materials prepared from small diameter h-BN, which is consistent with actual testing and simulation results.

### 3.3. Insulation Properties

**Figure 6:**
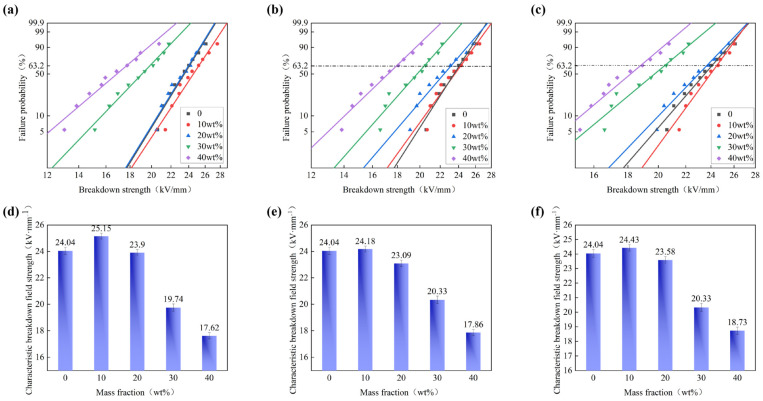
The AC breakdown strength of BMI-DBA/DMAA/h-BN composite materials. (**a**) 500 nm Weibull distribution chart; (**b**) 1 μm Weibull distribution chart; (**c**) 10 μm Weibull distribution chart; (**d**) 500 nm characteristic breakdown field strength; (**e**) 1 μm characteristic breakdown field strength; (**f**) 10 μm characteristic breakdown field strength.

In order to investigate the influence of h-BN distribution behavior on the insulation performance of BMI-DBA/DMAA, the AC breakdown field strength of BMI-DBA/DMAA/h-BN was tested, and Weibull distribution maps and characteristic breakdown field strength maps were drawn. The AC breakdown field strength of thermal conductive composite materials is shown in Figure 6, and the relevant parameters are shown in Table 3.

As shown in Figure 6, BMI-DBA/DMAA/h-BN prepared from h-BN of various scales showed a trend of increasing and then decreasing AC breakdown field strength with the increase of the h-BN filling amount. At a low filling amount (10 wt%), h-BN of all scales exhibited a phenomenon of higher AC breakdown field strength than pure BMI-DBA/DMAA. Because h-BN belongs to a wide bandgap and strong insulating inorganic particle, when a small amount of h-BN is added, the filler and BMI-DBA/DMAA resin tightly bond and interact at the interface between the two, hindering the migration of charge carriers and promoting the improvement of the electrical strength of the composite material. In the short-term breakdown process of solid dielectrics, breakdown develops in the form of electrical branches. When the electric branch contacts the inorganic filler, due to the high electrical strength of h-BN dispersed in the resin matrix, it can only “bypass” or “continue to develop along” the surface of h-BN, playing a certain role in the scattering of charge carriers, resulting in an increase in breakdown field strength [24]. However, high filling amount flake BMI-DBA/DMAA/h-BN composite materials exhibit relatively low electrical strength. Mainly attributed to the increase in viscosity of the resin system when the filler content is high, resulting in an increase in internal defects of the composite material, and the high addition of thermal conductive components hindering the curing degree of the resin. The introduction of a large amount of h-BN will form an “impurity bridge”. In a strong electric field environment, charge carriers can jump between fillers with continuous structures [25,26], which also reduces the insulation strength of the composite material to a certain extent.

**Figure 7:**
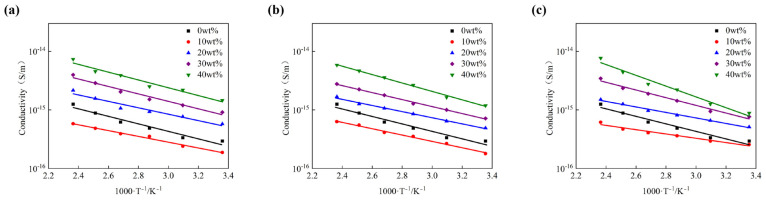
The variation of the electrical conductivity of BMI-DBA/DMAA/h-BN composites with the reciprocal of temperature. (**a**) 500 nm; (**b**) 1 μm; (**c**) 10 μm.

The variation of AC conductivity of BMI-DBA/DMAA/h-BN with temperature in the temperature range of 25–150 °C under the condition of an electric field strength of 10 kV/mm is shown in Figure 7.

As shown in Figure 7, the conductivity of BMI-DBA/DMAA/h-BN still exhibits a linear relationship with the reciprocal of absolute temperature. During the process of temperature increase, the macromolecular chains in BMI-DBA/DMAA tend to relax, and the amorphous region vibrates violently. The binding ability of the BMI-DBA/DMAA resin matrix to charge carriers weakens, resulting in an increase in charge carrier mobility and a significant increase in the conductivity of the composite material. From Figure 7, it can be seen that the addition of h-BN with diameters of 500 nm, 1 μm, and 10 μm leads to a decrease followed by an increase in the conductivity of BMI-DBA/DMAA. The addition of an appropriate amount of h-BN reduces the conductivity of BMI-DBA/DMAA resin, as the small-sized h-BN particles limit the migration of charge carriers within the BMI-DBA/DMAA resin, resulting in a decrease in the mobility of charge carriers in the composite material. When the mass fraction of filler exceeds 10 wt%, h-BN reduces the curing degree of BMI-DBA/DMAA resin through hydrogen bonding, leaving too many polar functional groups in the matrix that have not participated in the reaction, resulting in an increase in the conductivity of the composite material to a certain extent. When the addition amount is 40 wt%, the small-sized h-BN has a more significant effect on the conductivity increase of BMI-DBA/DMAA, mainly because the small-sized h-BN has a greater thickening effect on the resin, which can easily cause some defects in the cured material and affect the insulation performance of the composite material. The introduction of large-sized fillers generates a large number of interface voids, which have a negative impact on the performance of the insulation layer [27].

To investigate the temperature characteristics of BN/BMI-DBA conductivity, Equation (4) was used to fit the test results to obtain the activation energy *E* of the material’s conductivity:

(4) $$\ln\sigma \;=\;\ln\sigma_{0}\;-\;\frac{E}{kT}$$

where conductivity is represented by *σ*, *σ*_0_ is a parameter related to material properties, *k* is the Boltzmann constant, *E* is the activation energy of the sample (eV), which can reflect the sensitivity of conductivity to temperature changes, and *T* is the absolute temperature (K).

The results are shown in Table 4 for conductivity. According to Table 4, the activation energy of composite materials generally decreases first and then increases with the increase of filler content, mainly due to the increase of the interfacial layer inside the material caused by the appropriate addition of h-BN filler. As the temperature increases, the thickness of the interface layer gradually increases, thereby enhancing the scattering ability of charge carriers and increasing the inhibitory effect of the composite material on charge carrier migration [28]. However, excessive addition of h-BN reduces the crosslinking density of BMI-DBA/DMAA resin, loosens the three-dimensional network structure, and makes the molecular chain segments more prone to relaxation at high temperatures, resulting in increased temperature sensitivity of the composite material. The composite material filled with large-sized h-BN is more sensitive to temperature.

### 3.4. Dielectric Properties

**Figure 8:**
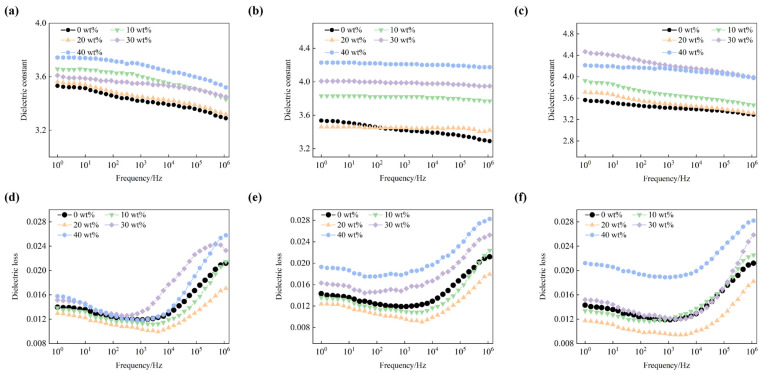
The dielectric properties of BMI-DBA/DMAA/h-BN composites. (**a**) 500 nm h-BN filling relative dielectric constant; (**b**) 1 μm h-BN filling relative dielectric constant; (**c**) 10 h-BN filling relative dielectric constant; (**d**) 500 nm h-BN fill the dielectric loss factor; (**e**) 1 μm h-BN fill the dielectric loss factor; (**f**) 10 μm h-BN fill the dielectric loss factor.

In order to investigate the effect of h-BN distribution on the dielectric properties of BMI-DBA/DMAA, the relative dielectric constant and dielectric loss factor of BMI-DBA/DMAA/h-BN were tested at room temperature. The relevant data are shown in Figure 8.

From Figure 8, it can be seen that the relative dielectric constant of the material shows a slight downward trend with increasing frequency, and its frequency dependence is relatively weak. This is mainly because as the frequency of the electric field changes, the interface polarization gradually becomes difficult to keep up with the speed of the applied electric field. The relative dielectric constant of BMI-DBA/DMAA and its composite materials is mainly determined by the displacement polarization of each component, the directional polarization of polar groups in the resin, and the interface polarization at the inorganic filler resin matrix interface. The three-dimensional network structure formed by the cured BMI-DBA/DMAA is tightly connected, making it difficult for polar groups to turn, resulting in a relatively low dielectric constant of BMI-DBA/DMAA. From the figure, it can be seen that for BMI-DBA/DMAA/h-BN prepared from h-BN of various scales, its relative dielectric constant shows an increasing trend with the increase of filling amount. This is because the relative dielectric constant of inorganic fillers is usually higher than that of BMI-DBA/DMAA, and the addition of inorganic fillers will construct more inorganic organic interfaces. In this interface, due to the difference in relative dielectric constants between the two materials, it will lead to an increase in the polarization degree of the composite material interface, resulting in a corresponding increase in the relative dielectric constant of the composite material. Under the same filler content, the relative dielectric constant of the composite material filled with nano BN is slightly lower than that of the composite material filled with micro flake BN. This is because the interface contact area of micrometer shaped h-BN is larger than that of nanometer shaped h-BN. When combined with BMI-DBA/DMAA resin, more inorganic organic interfaces are formed, and the polarization degree of the interface is stronger [29]. Therefore, the relative dielectric constant of micrometer shaped BMI-DBA/DMAA/h-BN resin composite material is higher.

From Figure 8, it can be seen that for BMI-DBA/DMAA/h-BN resin composite materials with different scales and filling amounts, their dielectric loss factors show a trend of decreasing first and then increasing with the frequency of the electric field. At low frequencies, the relaxation loss can be ignored, and the internal loss of the material system is mainly due to electrical conductivity loss. As the frequency continues to increase, relaxation polarization gradually becomes dominant in the material polarization process. With the continuous increase of frequency, relaxation polarization begins to lag behind the changes in the electric field, and the losses caused by relaxation polarization increase significantly, resulting in an increase in the dielectric loss factor of the material system. As the frequency increases, the directional migration of charge carriers inside the composite material decreases under the action of a higher frequency electric field, resulting in a decrease in conduction current and conductivity loss, thereby reducing the dielectric loss of the composite material. As the frequency continues to increase, the dielectric loss is mainly caused by the polarization of the polar groups of BMI-DBA/DMAA resin in the composite material. The addition of h-BN reduces the relative content of BMI-DBA/DMAA resin, and the dielectric loss caused by dipole motion of polymer molecules is significantly suppressed. Therefore, the dielectric loss factor of the composite material is lower than that of pure BMI-DBA/DMAA resin. In addition, considering the high degree of interface polarization in micro flake h-BN filled composite materials, their dielectric loss is slightly higher than that of nano h-BN filled composite materials.

### 3.5. Mechanical Properties

**Figure 9:**
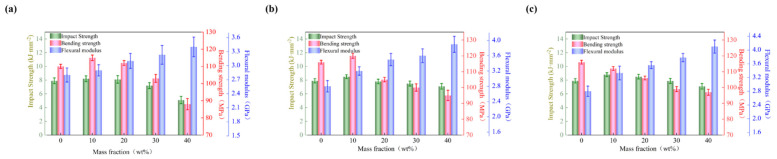
The mechanical properties of BMI-DBA/DMAA/h-BN composites. (**a**) 500 nm; (**b**) 1 μm; (**c**) 10 μm.

In order to investigate the influence of h-BN distribution characteristics on the mechanical properties of BMI-DBA/DMAA, the impact strength, bending strength, and bending modulus of BMI-DBA/DMAA/h-BN were tested as shown in Figure 9.

From Figure 9, it can be seen that the introduction of h-BN will cause the mechanical properties of the composite material to show a trend of first increasing and then decreasing, and the degree of decrease increases with the increase of h-BN addition. The reason for this phenomenon is that a small amount of h-BN introduction forms a winding structure with the polymer segments of the matrix resin, thereby improving the mechanical properties, while a large amount of h-BN addition will cause internal voids and a decrease in inverted mechanical properties.

**Figure 10:**
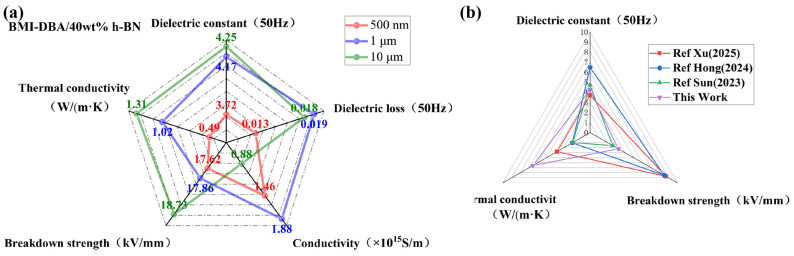
Performance comparison of BMI-DBA/DMAA/h-BN doped h-BN. (**a**) Performance comparison of BMI-DBA/DMAA/h-BN filled with h-BN of different diameters; (**b**) Comparison between BMI-DBA/DMAA/h-BN filled with 10 μm h-BN and related studies [30,31,32].

Figure 10 presents a summary and comparison of the performance of 40 wt% BMI-DBA/DMAA/h-BN filled with different sizes of h-BN. And a comparison between 10 μm diameter composite material and current research. From the comparative analysis in Figure 10, it can be seen that microscale h-BN has significant advantages in constructing thermal conductive networks. When the diameter size is 10 μm, the thermal conductivity of 40 wt% BMI-DBA/DMAA/h-BN reaches 1.31 W/(m·K), indicating that large-sized h-BN layers are more conducive to forming efficient three-dimensional thermal conductivity pathways. While maintaining excellent thermal conductivity, the 10 μm diameter composite material also exhibits superior insulation properties, with AC conductivity and breakdown field strength reaching 8.8 × 10^−16^ S/m and 18.73 kV/mm, respectively, significantly better than the small diameter filling system. Although the 10 μm filling system has relatively high relative dielectric constant (ε = 4.22) and dielectric loss factor (tan δ = 0.0197) in terms of dielectric properties, comparative analysis shows that the dielectric parameters of the three composite materials did not show significant changes. Based on a comprehensive evaluation of key indicators such as thermal conductivity, electrical insulation, and dielectric properties, the h-BN filling system with a wafer diameter of 10 μm exhibits the best electrical thermal synergistic performance.

## 4. Conclusions

The influence of the distribution pattern of h-BN on the electrical thermal properties of BMI-DBA/DMAA-based thermal conductive composite materials has been investigated: the thermal conductivity of the composite material increases with the increase of h-BN filling amount, among which h-BN with a sheet diameter of 10 μm has the most significant improvement in thermal conductivity. When the filling amount is 40 wt%, the thermal conductivity is 1.31 W/(m·K), and compared with other sizes of fillers, h-BN filled thermal conductive composite with a sheet diameter of 10 μm has excellent insulation performance.

## Figures and Tables

**Table 1:** The thermal conductivity of BMI-DBA/DMAA/h-BN.

Size	Mass Fraction (wt%)	80 °C Viscosity (mPa·s)	Thermal Conductivity *λ* (W/(m·K))	Upgrade Rate (*η*)
	0	980	0.19	0
Sheet diameter *l* = 500 nm Thickness *d* ≤ 200 nm	10	2013	0.22	15.79%
	20	4595	0.29	36.84%
	30	9806	0.34	78.95%
	40	18,207	0.49	157.89%
Sheet diameter *l* = 1 μm Thickness *d* ≤ 200 nm	10	1797	0.28	47.37%
	20	3282	0.37	94.74%
	30	6340	0.67	252.63%
	40	8120	1.02	436.84%
Sheet diameter *l* = 10 μm Thickness *d* ≤ 200 nm	10	1120	0.39	105.26%
	20	1753	0.69	263.16%
	30	2890	1.01	431.58%
	40	3945	1.31	589.47%

**Table 2:** BMI-DBA/DMAA/h-BN composite and the parameters of Y. Agari heat conduction model.

Size	C_1_	C_2_
Sheet diameter *l* = 500 nm Thickness *d* ≤ 200 nm	0.966	0.093
Sheet diameter *l* = 1 μm Thickness *d* ≤ 200 nm	1.055	0.156
Sheet diameter *l* = 10 μm Thickness *d* ≤ 200 nm	1.192	0.489

**Table 3:** Weibull parameters of the AC breakdown strength of BMI-DBA/DMAA/h-BN.

Size	Mass Fraction (wt%)	Eb (kV/mm)	β
	0	24.01	17.13
Sheet diameter *l* = 500 nm Thickness *d* ≤ 200 nm	10	25.15	13.82
	20	23.90	15.85
	30	19.74	10.72
	40	17.62	7.91
Sheet diameter *l* = 1 μm Thickness *d* ≤ 200 nm	10	24.18	13.51
	20	23.09	11.29
	30	20.33	12.12
	40	17.86	9.69
Sheet diameter *l* = 10 μm Thickness *d* ≤ 200 nm	10	24.43	18.48
	20	23.58	14.42
	30	20.33	12.12
	40	18.73	13.25

**Table 4:** The activation energy of BMI-DBA/DMAA/h-BN.

Size	Mass Fraction (wt%)	Activation Energy (eV)
	0	0.522
Sheet diameter *l* = 500 nm Thickness *d* ≤ 200 nm	10	0.426
	20	0.473
	30	0.548
	40	0.574
Sheet diameter *l* = 1 μm Thickness *d* ≤ 200 nm	10	0.467
	20	0.451
	30	0.515
	40	0.619
Sheet diameter *l* = 10 μm Thickness *d* ≤ 200 nm	10	0.316
	20	0.405
	30	0.566
	40	0.793

## Data Availability

The original contributions presented in this study are included in the article. Further inquiries can be directed to the corresponding authors.
